# Sociodemographic predictors of public attitudes toward deceased organ donation and organ trade in Kazakhstan

**DOI:** 10.3389/fsoc.2026.1755804

**Published:** 2026-06-26

**Authors:** Akbota Kassymova, Assem Omarova

**Affiliations:** 1 Department of Sociological Science and Social Work, Al-Farabi Kazakh National University, Almaty, Kazakhstan; 2 Department of General Education, Egyptian University of Islamic Culture Nur-Mubarak, Almaty, Kazakhstan

**Keywords:** cross-country comparison, deceased organ donation, education level, Kazakhstan, organ trafficking, public attitudes, sociological survey

## Abstract

Public attitudes toward deceased organ donation emerge at the intersection of cultural norms, moral beliefs, media narratives, and trust in health institutions. In post-Soviet contexts such as Kazakhstan, low levels of health literacy and persistent rumors surrounding a “black market” in organs continue to function as barriers to donation and as sources of skepticism toward medical institutions. Despite recent legislative reforms and public-awareness initiatives, sociological evidence explaining how demographic, cognitive, and institutional factors shape these perceptions remains limited. This study examines how age and education influence public attitudes toward deceased organ donation and beliefs about illegal organ trade in Kazakhstan, while also exploring behavioral readiness for formal donor registration and the hierarchical interaction of cognitive and institutional determinants of donation attitudes. Between February 2024 and April 2025, a representative survey of 569 adult residents of Almaty and the Almaty region was conducted using a quota-based sample stratified by age, gender, and education level. Descriptive statistics and Fisher’s Exact Test were used to examine demographic differences in awareness and trafficking-related beliefs, while multivariable logistic regression and a decision-tree classifier were applied to evaluate behavioral readiness for donor registration and hierarchical relationships among cognitive, institutional, and attitudinal predictors. Analyses were performed using IBM SPSS v.28 and Python (v.3.10). Education level emerged as the strongest and most consistent predictor of informed and critical attitudes toward deceased organ donation. Respondents with higher education were more likely to demonstrate awareness of legal and medical donation criteria and less likely to endorse trafficking-related misconceptions. Age primarily differentiated preferred communication channels, with younger respondents relying more frequently on digital media and older groups preferring television-based information sources, while no statistically significant association was observed between age and belief in organ trafficking. The adjusted regression analysis additionally indicated that place of residence was the strongest independent predictor of willingness to formally register donor consent through the national e.gov system. The decision tree analysis identified brain-death awareness and institutional trust as the central hierarchical determinants shaping support for deceased organ donation. The findings suggest that public attitudes toward organ donation in Kazakhstan are shaped not only by demographic characteristics but also by broader institutional and informational environments. Strengthening health literacy, improving institutional transparency, and developing communication strategies adapted to different social groups may increase trust, reduce misinformation, and enhance public readiness to support deceased organ donation and formal donor registration.

## 1 Introduction

Organ transplantation occupies a unique position at the intersection of medicine, ethics, and social trust. Beyond its clinical importance, it embodies a moral relationship between individuals and society-a voluntary exchange that transforms the human body into a symbol of solidarity. As such the decision to donate organs after death is never purely medical; it reflects how people interpret life, mortality, and moral obligation. Public attitudes therefore depend on a delicate balance of cultural beliefs, religious traditions, and confidence in institutions responsible for transplantation.

Globally, the success of organ-donation programs hinges on institutional transparency, legal clarity, and public participation. Comparative studies show that nations with sustained awareness campaigns and robust coordination systems-such as Spain, the Netherlands, and the United Kingdom-achieve high post-mortem donation rates (Matesanz and Domínguez-Gil, 2007; López-Fraga et al., 2014). Conversely, when public trust is fragile or misinformation dominates the media space, willingness to donate declines sharply (Rithalia et al., 2009). The sociology of health therefore treats organ donation as a social process mediated by information flows, moral norms, and governance capacity rather than a purely biomedical practice. Recent empirical research further demonstrates that institutional trust and perceived transparency of organ allocation systems remain central predictors of willingness to donate in middle-income countries (Baspinar et al., 2024).

In Kazakhstan this issue remains controversial. The country has introduced a digital registry for consent and established a national coordination center, yet participation rates remain extremely low. Official data show that the number of refusals exceeds consents by roughly fifteen to one (Bolatov et al., 2025a). Rumors about a “black market” in human organs, amplified through social networks and informal conversations, continue to fuel distrust toward both medical institutions and the legal system. Recent studies highlight the growing role of misinformation and digital rumor ecosystems in shaping public trust in health systems across post-Soviet societies (Bolatov et al., 2025b; Sazonov et al., 2025), while global research has also documented how organ-trafficking narratives influence public perceptions of transplantation systems (Budiani-Saberi and Columb, 2013; Meyer, 2006). Consistent with these patterns, national evidence indicates that generally supportive attitudes toward organ donation often coexist with reluctance to provide formal consent, reflecting a behavioral gap driven by institutional distrust and perceived procedural opacity (Bolatov et al., 2025b; Sazonov et al., 2025).

Although governmental and religious leaders have called for public dialogue, comprehensive sociological research on what actually shapes these attitudes is scarce. Most existing analyses focus on legal regulation or clinical outcomes, leaving cultural and cognitive factors underexplored (Zhetpisbaeva and Tazhibaeva, 2014). Earlier survey evidence from Kazakhstan also confirms substantial variability in public opinion regarding post-mortem donation, highlighting the importance of demographic stratification in attitude analysis (Doskhan et al., 2020).

Religion may also represent an important component of public discourse surrounding organ donation in Kazakhstan, where public attitudes toward transplantation are shaped by broader informational and institutional factors, including medical mistrust and misinformation (Bolatov et al., 2025b; Sazonov et al., 2025). Although the present study did not include direct measures of religiosity or religious affiliation, several questionnaire items contained response categories related to religious beliefs and religious organizations. Similar patterns have also been discussed in studies conducted in other Muslim-majority societies, where religious interpretation may influence public acceptance of transplantation practices (Yilmaz, 2011; Baspinar et al., 2024). Accordingly, religion in the present study was considered indirectly as part of the broader normative and communication environment surrounding deceased donation.

International evidence suggests that sociodemographic variables-particularly education and age-strongly influence how individuals perceive ethical issues in medicine. Education enhances health literacy and critical thinking, enabling citizens to differentiate factual information from rumor (Yilmaz, 2011; Hyde and White, 2009). Recent public-health research also highlights the growing role of misinformation dynamics and digital media exposure in shaping organ-donation attitudes, particularly in transitional information environments. Comparative studies similarly indicate that higher educational attainment and socioeconomic resources significantly increase willingness to consent to deceased donation across several middle-income countries (Rasiah et al., 2014; Barcellos et al., 2005; Al-Jumah and Abolfotouh, 2011).

Age affects both value orientations and preferred media channels, shaping exposure to moral discourse (Sanner, 2006; Kobus et al., 2016; Afshar et al., 2012). However, these relationships have not been tested empirically within the post-Soviet sociocultural setting, where generational experiences differ sharply and institutional communication remains uneven. Understanding their relative impact is therefore essential for evidence-based health policy.

Theoretically, the study draws on sociological perspectives that interpret organ donation as a moral and institutional process shaped by trust, governance structures, and perceived fairness of medical systems within. In this perspective, attitudes toward organ donation reflect how societies negotiate the boundaries between altruism, individual autonomy, and institutional responsibility, situating donation practices within broader moral economies of health and inequality (Scheper-Hughes, 2000). Applying this framework to Kazakhstan allows interpretation of local skepticism not merely as a consequence of limited awareness but also as a rational response to broader contexts of institutional trust, governance quality, and perceived corruption. This approach aligns with the health-literacy paradigm, which links informed consent to individuals’ capacity to access, understand, and apply medical information (Rademakers et al., 2022). Combining these perspectives provides an interdisciplinary lens that integrates sociology, ethics, and public health in the analysis of donation attitudes. Together, recent empirical studies suggest that demographic structure, institutional trust, and health literacy jointly shape donation attitudes, reinforcing the need for multidimensional sociological modelling in post-Soviet contexts (Bolatov et al., 2025b; Sazonov et al., 2025).

Over the past 5 years, recent studies indicate that the effectiveness of healthcare engagement increasingly depends on the interplay between health literacy, digital communication environments, and institutional trust. Evidence suggests that targeted health literacy interventions can mitigate the effects of medical misinformation, whereas fragmented information ecosystems may undermine public confidence in healthcare systems. However, empirical analyses that jointly examine these mechanisms remain limited in post-Soviet contexts, underscoring the need for context-specific evidence (Rademakers et al., 2022; Chen et al., 2023; Bolatov et al., 2025b; Sazonov et al., 2025).

Empirically, the present research fills three major gaps. First, no prior quantitative study in Central Asia has modeled how demographic variables predict attitudes toward both deceased organ donation and the illegal organ trade. Second, previous surveys have treated age and education primarily as background descriptors rather than explanatory predictors of awareness, trust, and behavioral readiness. Third, cross-cultural comparisons seldom consider the hybrid information environment of post-Soviet societies, where digital media, state messaging, and informal rumor coexist.

Addressing these gaps, this study explores how age and education jointly shape public attitudes toward organ donation in Kazakhstan and evaluates whether belief in the existence of an illegal organ market varies across demographic groups. Using a representative survey of 569 adults in Almaty and the Almaty region, the study combines conventional statistical analysis with multivariable and decision-tree modelling approaches in order to examine both independent associations and hierarchical interaction structures among demographic, cognitive, and institutional predictors. This analytical framework not only quantifies associations but also visualizes conditional decision pathways shaping support for deceased organ donation and behavioral readiness for formal donor registration.

The relevance of this research extends beyond Kazakhstan. Many middle-income countries face similar challenges, including low institutional trust, uneven media literacy, fragmented communication environments, and conflicting moral narratives surrounding transplantation (Da Silva and Frontera, 2015). Understanding how demographic, cognitive, and institutional factors interact under these conditions may help inform international strategies for ethical organ procurement and publichealth communication. From a sociological perspective, the findings highlight how modern citizenship is negotiated through moral responsibility for the body, trust in science, and participation in collective health initiatives.

Conceptually, the study distinguishes between several interconnected dimensions of public engagement with deceased organ donation, including general awareness, factual knowledge, normative attitudes, and behavioral readiness for formal donor registration. Beyond examining demographic differences in awareness and perceptions of organ trafficking, the study also explores how cognitive and institutional factors jointly shape support for deceased organ donation and willingness to translate supportive attitudes into formal registration behavior.

Accordingly, this study pursues three objectives:

1. To determine whether age cohorts differ significantly in their perceptions of deceased organ donation and organ trafficking.
2. To assess how education level predicts awareness and critical reasoning.
3. To evaluate how demographic, cognitive, and institutional factors jointly shape attitudes toward deceased organ donation and behavioral readiness for donor registration using multivariable and decision-tree modelling approaches.

By integrating empirical evidence with theoretical reflection, the article contributes to the global discussion on health literacy, moral responsibility, and social inequality in transplantation. It argues that improving public understanding of organ donation requires not only legal reform but also investment in education, transparent communication, and trust-building between citizens and medical institutions. In doing so, it reframes deceased organ donation as a test of collective ethics and social cohesion in the era of digital information.

## 2 Materials and methods

### 2.1 Research design and rationale

This research employed a cross-sectional sociological survey conducted in Almaty and Almaty region (Kazakhstan) between February 2024 and April 2025.

A quantitative design was selected to identify demographic predictors of attitudes toward deceased organ donation and beliefs about organ trafficking (Creswell, 2013). The city of Almaty and the Almaty region were selected as the empirical setting of the study, as together they constitute the largest population agglomeration and one of the most socio-culturally diverse macro-regions in Kazakhstan. This territorial configuration enables the capture of both metropolitan dynamics and adjacent peri-urban transformations, thereby serving as a robust proxy for broader national urban and regional trends, while simultaneously ensuring logistical feasibility and operational efficiency for fieldwork implementation.

To contextualize national data, the study additionally incorporated a comparative analysis of public-donation indicators from four countries-Spain, Turkey, Russia, and China**-**representing distinct models of transplantation governance. Spain was included as a global benchmark of institutional success; Turkey and Russia share cultural and religious similarities with Kazakhstan; China exemplifies a rapidly developing system transitioning from ethically controversial to regulated donation practices. This selection allows for analytical triangulation between high-trust, hybrid, and low-trust health-system contexts.

### 2.2 Sampling and participants

The initial dataset consisted of 581 respondents. During data cleaning, 12 questionnaires were excluded due to incomplete responses and inconsistencies. The final analytical sample therefore comprised 569 respondents. The target population consisted of adults aged 18–62 years residing in Almaty and Almaty region; respondents over 62 were excluded in accordance with Kazakhstani legal and medical donation criteria. A quota-based sampling strategy was employed to ensure proportional representation of respondents by age, gender, and education, based on the demographic structure of the city population. Data collection was conducted between February 2024 and April 2025 using a mixed-mode approach that combined an online survey with face-to-face questionnaire administration. Participants were recruited through social media platforms, university communities, and public spaces across the city. Prior to the main fieldwork, the questionnaire was pilot-tested on a sample of 25 participants to assess clarity and sequencing of the items.

To enhance transparency and replicability, additional procedural details of the quota implementation are provided. Quotas for age, gender, and education were calculated prior to fieldwork using official demographic statistics for Almaty and the Almaty region. A monitoring table was maintained throughout the data-collection period. The field coordinator reviewed quota fulfillment weekly. Recruitment within a specific stratum was discontinued once the predefined target for that category was reached.

To minimize duplication in the online component, the survey platform restricted multiple submissions from the same IP address and device. Responses with identical demographic profiles and completion timestamps were additionally screened and removed during data cleaning. No automated or bot-generated entries were detected after verification of response patterns and completion times.

Data collection followed a mixed-mode design. Approximately 58% of responses were obtained online and 42% through face-to-face administration in public and institutional settings. The questionnaire content and structure were identical across modes.

Post-stratification weighting was not applied. The achieved sample closely matched the predefined quota structure across age, gender, and education categories. Minor deviations remained within 3% points and were not considered sufficient to justify statistical weighting. Therefore, unweighted data were used in all analyses.

### 2.3 Questionnaire and variables

The questionnaire consisted of 19 items grouped into four thematic domains:

1. Socio-demographic characteristics (age, gender, education, place of residence, social status, and income).
2. Awareness and attitudes toward organ donation (knowledge of legal donation criteria, perceptions of brain death and post-mortem donation, willingness to consent to organ donation, and trust-related perceptions regarding transplantation practices).
3. Perceptions of illegal organ trade and institutional inequality in transplantation access.
4. Information channels and communication environments related to organ donation (digital media, television, healthcare professionals, educational institutions, and religious organizations).

Place of residence was included as a contextual socio-demographic variable and categorized into two groups: respondents residing in Almaty city and respondents residing in urban or rural settlements of the Almaty region.

The awareness domain included both self-reported awareness items and factual knowledge questions concerning donor registration procedures, waiting lists, legal mechanisms of postmortem donation, and medically certified brain-death-related transplantation practices in Kazakhstan.

General awareness of deceased organ donation was operationalized as a binary variable indicating whether respondents had previously heard about post-mortem organ donation.

In addition, the ability to correctly identify medical criteria for post-mortem donation was assessed through factual questionnaire items related to brain death and legally regulated donation procedures in Kazakhstan. Respondents were classified as demonstrating correct medical knowledge if they correctly identified at least one legally valid mechanism of post-mortem organ donation, including consent registered through the e.gov portal or donation following a medically certified diagnosis of brain death. All other responses were coded as incorrect or uncertain.

Normative attitudes toward deceased donation were assessed through questions concerning moral acceptability of organ transplantation, willingness to become a donor, attitudes toward post-mortem donation, and perceptions of donor inequality and illegal organ trade. The communication domain examined both primary sources of information about organ donation and socially legitimate channels for public-awareness campaigns.

Although the questionnaire did not include direct indicators of religiosity or religious affiliation, several response categories indirectly reflected the potential role of religion in shaping attitudes toward organ donation. In particular, religion-related response options appeared in questions concerning sources of information about transplantation, moral acceptability of deceased donation, and public-awareness initiatives involving religious organizations.

The questionnaire employed dichotomous, multiple-choice, and ordinal response formats, including five-point Likert scales ranging from 1 (“strongly disagree”) to 5 (“strongly agree”). Several items additionally included “difficult to answer” response categories in order to reduce forced-choice bias and capture uncertainty in public attitudes toward transplantation.

For analytical purposes, selected categorical responses were recoded into binary or ordinal indicators suitable for descriptive analysis, logistic regression, and decision-tree modelling. All questionnaire items were developed in Russian and Kazakh and pre-tested for linguistic clarity and respondent comprehension prior to data collection.

All predictors included in the decision-tree model were derived exclusively from the primary survey data collected through the study questionnaire. Unlike the contextual crossnational analysis, which incorporated external international datasets, the predictive modelling component relied solely on variables operationalized from the original survey instrument. “Brain Death Awareness” was constructed from respondents’ knowledge regarding transplantation waiting lists (Question 7.3), legal mechanisms of post-mortem organ donation, and medically certified brain-death procedures (Question 9 and 15). “Digital Government Trust” reflected respondents’ trust in official institutional information sources (Question 8) and willingness to register donor consent through the national e.gov portal (Question 14). “Ethical Acceptability of Deceased Donation” was derived from attitudes toward the permissibility of organ retrieval after brain death (Question 9) and personal willingness to become an organ donor (Question 10). “Perceived Institutional Inequality” was constructed from responses concerning perceived unfairness in transplantation access, including beliefs regarding illegal organ trade and commercialization risks in transplantation practices (Question 12 and 13).

During data preparation, categorical responses were recoded into binary indicators, whereas the outcome variable representing willingness toward organ donation was retained as a multiclass variable (negative/undecided/positive). The full questionnaire is provided in the Supplementary material (Appendix A), while the operationalization of analytical variables and their correspondence to questionnaire items are summarized in Appendix D.

### 2.4 Comparative dataset and indicator selection

To strengthen the sociological interpretation of the national findings, we complemented the survey data with an international comparative dataset for the period 2018–2024. Secondary statistics were retrieved from the WHO Global Observatory on Donation and Transplantation (World Health Organization (WHO) and Spanish National Transplant Organization (ONT), 2024).

For each country, we selected a small set of indicators that capture both the quantitative and institutional dimensions of organ donation. These included: (1) the number of deceased donors per million population (pmp), (2) the proportion of post-mortem versus living donations, and (3) proxy measures of institutional trust and the intensity of public-awareness campaigns, as reported in peer-reviewed studies and official reports. Together, these indicators provide a basis for situating Kazakhstan within different governance models of transplantation and for interpreting attitudinal patterns observed in our survey against broader international trends.

Cross-national comparisons were conducted using publicly available indicators of transplantation system performance reported by international registries and peer-reviewed studies. No composite institutional trust index was constructed in this study. Instead, a descriptive benchmarking framework was applied, comparing countries using three groups of observable indicators: (1) transplantation system performance indicators (deceased donors per million population), (2) governance characteristics of transplantation systems (legal consent model, institutional coordination structure), and (3) public communication and engagement indicators (reported national awareness campaigns, documented public outreach activity, and observed awareness trends reported in international and national reports).

Countries were descriptively categorized according to the convergence of these indicators using data from the WHO Global Observatory on Donation and Transplantation, WHO transplantation system reports, and recent peer-reviewed empirical studies (Table 1). This benchmarking strategy ensures methodological transparency by relying exclusively on internationally documented indicators and avoids potential subjectivity associated with constructing weighted composite trust indices, while enabling contextual comparison of national transplantation environments.

**Table 1:** Comparative overview of organ-donation systems, 2018–2024.

Country	Donors per million population (2024)	Main governance model	Public awareness trend
Spain	46.3 pmp	Centralized opt-out system with strong public trust	High engagement, stable positive media
Turkey	11.2 pmp	Hybrid opt-in system with religious endorsement by Diyanet	Moderate awareness, religious support essential
Russia	4.6 pmp	De facto opt-out, low institutional transparency	Low trust, limited campaigns
China	6.8 pmp	State-regulated opt-in system (post-2015 reforms)	Rapid growth, strong media presence
Kazakhstan	3.9 pmp	Opt-out by law, but opt-in in practice	Weak public trust, fragmented information

Sources: Data were retrieved from the Global Observatory on Donation and Transplantation (World Health Organization (WHO) and Spanish National Transplant Organization (ONT), 2024; Bolatov et al., 2025b).

The table presents descriptive benchmarking indicators of transplantation system performance, governance arrangements, and publicly documented public-awareness dynamics across selected countries. Donor rates (per million population) are based on the most recent available international registry data, while governance characteristics and awareness trends were compiled from WHO transplantation system reports, national transplantation authority publications, and peer-reviewed comparative studies. No composite institutional trust index was constructed; country comparisons rely solely on observable international indicators.

Based on these observable benchmarking indicators, Kazakhstan is positioned among transplantation systems characterized by comparatively lower donor rates and less intensive public-awareness communication environments, where formal legal consent frameworks are present but awareness and coordination indicators remain comparatively limited. Spain represents a highly coordinated transplantation system with sustained public communication strategies; Turkey illustrates a culturally proximate context where institutional religious endorsement supports donation awareness; Russia provides a post-Soviet institutional comparison with similar coordination constraints; and China demonstrates the role of large-scale government-led awareness campaigns in recent system expansion.

### 2.5 Analytical procedures

Data were processed using IBM SPSS v.28 and Python (v.3.10).

1. Descriptive statistics were used to summarize sample characteristics, response distributions, and the prevalence of awareness, knowledge, and attitudes toward post-mortem organ donation.
2. Fisher’s Exact Test was applied to examine associations between categorical sociodemographic variables (primarily age, education level, and place of residence) and key attitudinal indicators, including awareness of organ donation, knowledge of legal and medical donation criteria, and belief in organ-trafficking narratives (Field, 2013).
3. In addition to the attitudinal analyses, a supplementary multivariable logistic regression model was estimated to examine behavioral readiness for formal registration of consent for postmortem organ donation through the national e.gov system. While the primary focus of the study concerned public attitudes toward deceased organ donation and organ trafficking, registration behavior represents a more institutionally embedded form of participation reflecting willingness to translate attitudes into formal consent. This additional analysis was included to evaluate whether demographic characteristics associated with awareness and trust were also related to actual registration intentions.

The dependent variable was registration of consent for post-mortem organ donation (1 = willing to register consent via e.gov; 0 = refusal or uncertainty). Independent variables included age, gender, education level, income, and place of residence. Education, income, and residence were treated as categorical variables and dummy coded. The reference categories were secondary education, no income, and residence in Almaty city, respectively. Age was entered as a continuous predictor using interval midpoints. Robust standard errors (HC0) were applied due to the relatively low prevalence of positive registration outcomes. Model estimates are reported as adjusted odds ratios (AORs) with 95% confidence intervals.

1 To explore non-linear and hierarchical relationships among cognitive, institutional, and ethical determinants of donation attitudes, a Decision Tree Classifier was constructed using predictors including brain-death awareness, digital government trust, perceived social inequality, and post-mortem acceptability. Unlike the regression analysis, which examined independent associations with formal donor registration, the decision-tree approach was intended primarily as an exploratory and interpretative framework for identifying conditional interaction structures and threshold effects among attitudinal predictors. This approach enabled visualization of sequential decision pathways shaping support for post-mortem organ donation while providing interpretable hierarchical patterns of cognitive and institutional influence.

**Figure 1:**
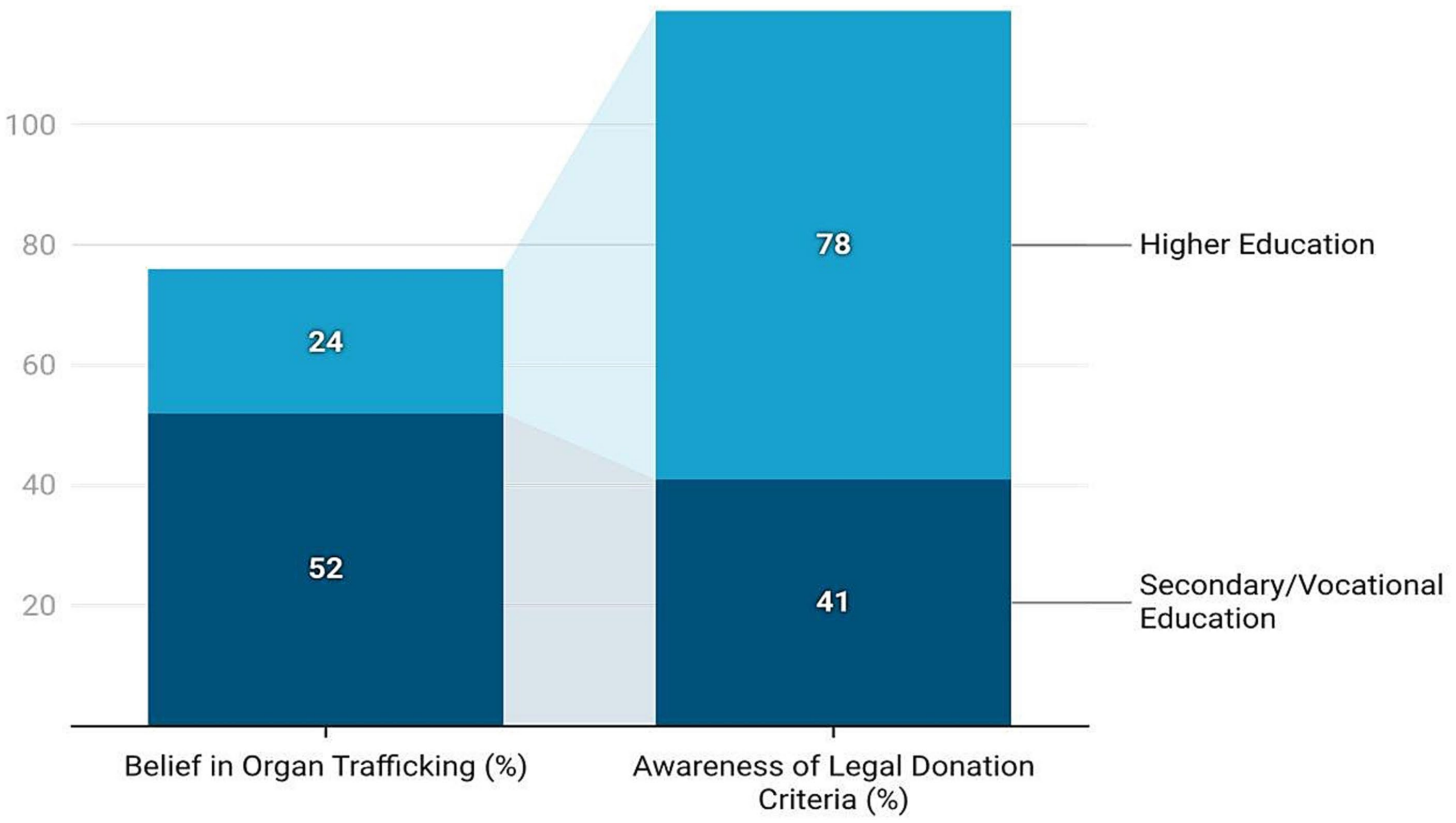
Differences in awareness of legal donation criteria and belief in organ trafficking according to education level. Source: authors’ analysis (2025).

Figure 1 illustrates differences in awareness of legal donation criteria and belief in organ trafficking according to education level, highlighting higher awareness and lower endorsement of trafficking-related beliefs among respondents with higher education.

### 2.6 Ethical considerations

The study followed the *Declaration of Helsinki (2013 revision)* and was approved by the Institutional Review Board of Al-Farabi Kazakh National University (IRB-A417, 31 March 2022).

All participants provided informed consent and were assured of anonymity and voluntary participation. No incentives were given. The comparative international data are publicly accessible and did not involve human subjects.

### 2.7 Comparative interpretation framework

The cross-national component serves two analytical purposes:

2 To benchmark Kazakhstan against countries that illustrate distinct models of policy effectiveness.
3 To reveal how variations in religious authority, state capacity, and information governance mediate public trust.

In Spain, centralized coordination and consistent communication yield high trust and high donor rates.

In Turkey, religious legitimization compensates for limited administrative capacity, demonstrating how ethical endorsement and trust in intermediaries can increase acceptance of organ donation (Yilmaz, 2011; Baspinar et al., 2024).

In Russia, despite similar legal structures to Kazakhstan, limited public trust and insufficient institutional communication continue to constrain post-mortem donation participation.

China demonstrates how sustained government media campaigns, after the 2015 reform banning organ sourcing from executed prisoners, significantly raised voluntary registration (Chen et al., 2023).

These contrasts help interpret Kazakhstan’s stagnation not as cultural fatalism but as a communication deficit combined with moderate health literacy.

### 2.8 Summary of methodological strengths and limitations

The main strength lies in combining representative survey data with comparative international benchmarks and advanced analytical techniques. The study’s limitations include its territorial concentration within the city of Almaty and the Almaty region, which may constrain the generalizability of the findings to other regional contexts. Nevertheless, the quantitative rigor and contextualization across similar sociocultural settings enhance the external validity of the findings.

## 3 Results

### 3.1 Descriptive results of the survey

Among 569 respondents, 63% reported having heard about deceased organ donation (general awareness), whereas only 21% were able to correctly identify its medical or legal criteria (factual knowledge). This indicates a substantial gap between general awareness and substantive understanding of the donation process. While a majority of respondents were familiar with the concept of organ donation, only a minority demonstrated accurate operational knowledge of its medical and legal foundations.

Table 2 presents the socio-demographic structure of the sample together with selected attitudinal indicators related to deceased organ donation, knowledge of donation criteria, and registration readiness. The achieved sample demonstrated substantial variation across sociodemographic characteristics and attitudinal dimensions, thereby supporting the analytical objectives of the study.

**Table 2:** Socio-demographic and selected attitudinal characteristics of respondents.

Characteristic	Categories	% of sample (*n* = 569)
Age	18–35 yrs. (*n* = 281); 36–44 yrs. (*n* = 112); 45–62 yrs. (*n* = 176)	49.4 19.7 30.9
Gender	Women (*n* = 307); Men (*n* = 262)	54 46
Education	Higher (*n* = 341); Incomplete higher (*n* = 53); Vocational (*n* = 78); Secondary (*n* = 97)	59.9 9.3 13.7 17.0
Income	No income (*n* = 146); ≤250,000 ⫧ (*n* = 191); 250–360,000 ⫧ (*n* = 137); 370–500,000 ⫧ (*n* = 64) >500,000 ⫧ (*n* = 31)	25.7 33.6 24.1 11.2 5.4
Place of residence	Almaty city (republican city) (*n* = 200); Urban and rural settlements within the Almaty region (*n* = 369)	35.1 64.9
General awareness of deceased organ donation	Aware of deceased donation (*n* = 358); Not aware (*n* = 211)	63 37
Ability to correctly define medical donation criteria	Correctly identified medical criteria (*n* = 119); Incorrect/uncertain responses (*n* = 450)	21 79
Registered consent for post-mortem organ donation	Yes (*n* = 34); No (*n* = 535)	5.9 94.1

Source: authors’ survey (2025).

Overall, 63% of respondents reported general awareness of deceased organ donation, whereas only 21% were able to correctly identify the medical and legal criteria for post-mortem donation. Only 5.9% reported willingness to formally register donor consent through the national e.gov system. In addition to awareness-related indicators, the survey also examined normative attitudes toward deceased organ donation and behavioral willingness to support donation practices. Overall, supportive attitudes toward post-mortem donation were substantially more common than readiness for formal donor registration, suggesting the presence of a gap between general moral acceptance of donation and willingness to translate these attitudes into institutionalized behavioral commitment.

Table 3 summarizes differences in awareness and trafficking-related beliefs across education groups.

**Table 3:** General awareness of deceased organ donation and belief in organ trafficking by education level.

Education level	General awareness of deceased organ donation (%)	Belief in organ trafficking (%)
Higher	78 (*n* = 266)	24 (*n* = 82)
Incomplete higher	59 (*n* = 31)	37 (*n* = 20)
Vocational	48 (*n* = 37)	45 (*n* = 35)
Secondary	41 (*n* = 40)	52 (*n* = 50)

Source: authors’ survey (2025).

Respondents with higher education were more likely to report general awareness of deceased organ donation (78%) and less likely to believe in organ-trafficking rumors (24%). In contrast, respondents with secondary education demonstrated lower awareness (41%) and higher endorsement of trafficking-related beliefs (52%). Fisher’s Exact Test confirmed a statistically significant association between education level and awareness of deceased organ donation (*p* < 0.01), indicating that education represented the strongest socio-demographic correlate of informed donation-related attitudes within the sample.

Age-related differences were substantially weaker. Younger respondents demonstrated slightly higher self-reported awareness of donation-related information and relied more frequently on digital media sources, whereas older respondents more often preferred television based information channels. However, no statistically significant association was observed between age and belief in organ trafficking (*p* = 0.43).

Additional exploratory analyses examined whether place of residence was associated with awareness of deceased organ donation and trafficking-related beliefs. Respondents residing in urban and rural settlements of the Almaty region demonstrated slightly higher levels of awareness and greater readiness to consider donor registration compared to respondents from Almaty city; however, associations with trafficking-related beliefs remained weak and statistically inconsistent.

### 3.2 Multivariable logistic regression analysis

As a supplementary behavioral analysis, the multivariable logistic regression model examined factors associated with formal registration of consent for post-mortem organ donation through the national e.gov system. Place of residence was the only statistically significant independent predictor after adjustment for age, gender, education, and income (Table 4). Respondents residing in urban and rural settlements of the Almaty region demonstrated significantly higher odds of registration compared to respondents residing in Almaty city (AOR = 4.28; 95% CI: 1.53–11.94; *p* = 0.005). Higher education and higher income levels demonstrated positive but statistically borderline associations with willingness to register consent for post-mortem organ donation, whereas age and gender were not independently associated with registration after adjustment (Appendix B).

**Table 4:** Multivariable logistic regression analysis of factors associated with registration of consent for deceased organ donation.

Variable	AOR	95% CI	*p*-value
Higher education	4.29	0.93–19.68	0.061
Residence in urban/rural settlements of the Almaty region	4.28	1.53–11.94	0.005
High-income category (>500,000 ⫧)	3.06	0.85–11.07	0.087
Age (continuous)	1.01	0.98–1.04	0.421
Female gender	0.47	0.20–1.10	0.084

Source: authors’ survey (2025).

Adjusted for age, gender, education level, income, and place of residence. Reference categories were secondary education, no income, and residence in Almaty city.

Across all descriptive and inferential analyses, education consistently demonstrated a stronger association with awareness of donation criteria and belief in organ trafficking compared to age. While age showed no statistically significant association with belief in trafficking (*p* = 0.43), education was significantly associated with awareness (*p* < 0.01), indicating its relatively greater explanatory relevance within the observed patterns.

### 3.3 Decision-tree model

Building upon the descriptive and behavioral findings presented in Sections 3.1 and 3.2, the decision-tree analysis was used to explore how cognitive, institutional, and ethical factors interact hierarchically in shaping support for deceased organ donation. To examine these conditional interaction structures, a theory-informed Decision Tree classifier was developed using predictors related to brain-death awareness, institutional trust, perceived social inequality, and post-mortem acceptability.

The decision-tree analysis revealed a hierarchical structure of cognitive, institutional, and ethical determinants shaping attitudes toward deceased organ donation. Brain-Death Awareness emerged as the primary predictor, indicating that respondents who correctly understood the medical and legal criteria of brain death were substantially more likely to support deceased organ donation. This finding suggests that health literacy may function as a key cognitive threshold separating informed acceptance from uncertainty and misinformation.

The model further demonstrated that positive attitudes toward organ donation were more common among respondents exhibiting higher institutional trust and greater acceptance of postmortem donation practices. In particular, trust in digital government mechanisms and official transplantation procedures was associated with greater willingness to consider formal donor consent. Conversely, perceptions of social inequality and concerns regarding unfair access to transplantation resources were associated with greater hesitation and skepticism toward the donation system.

**Figure 2:**
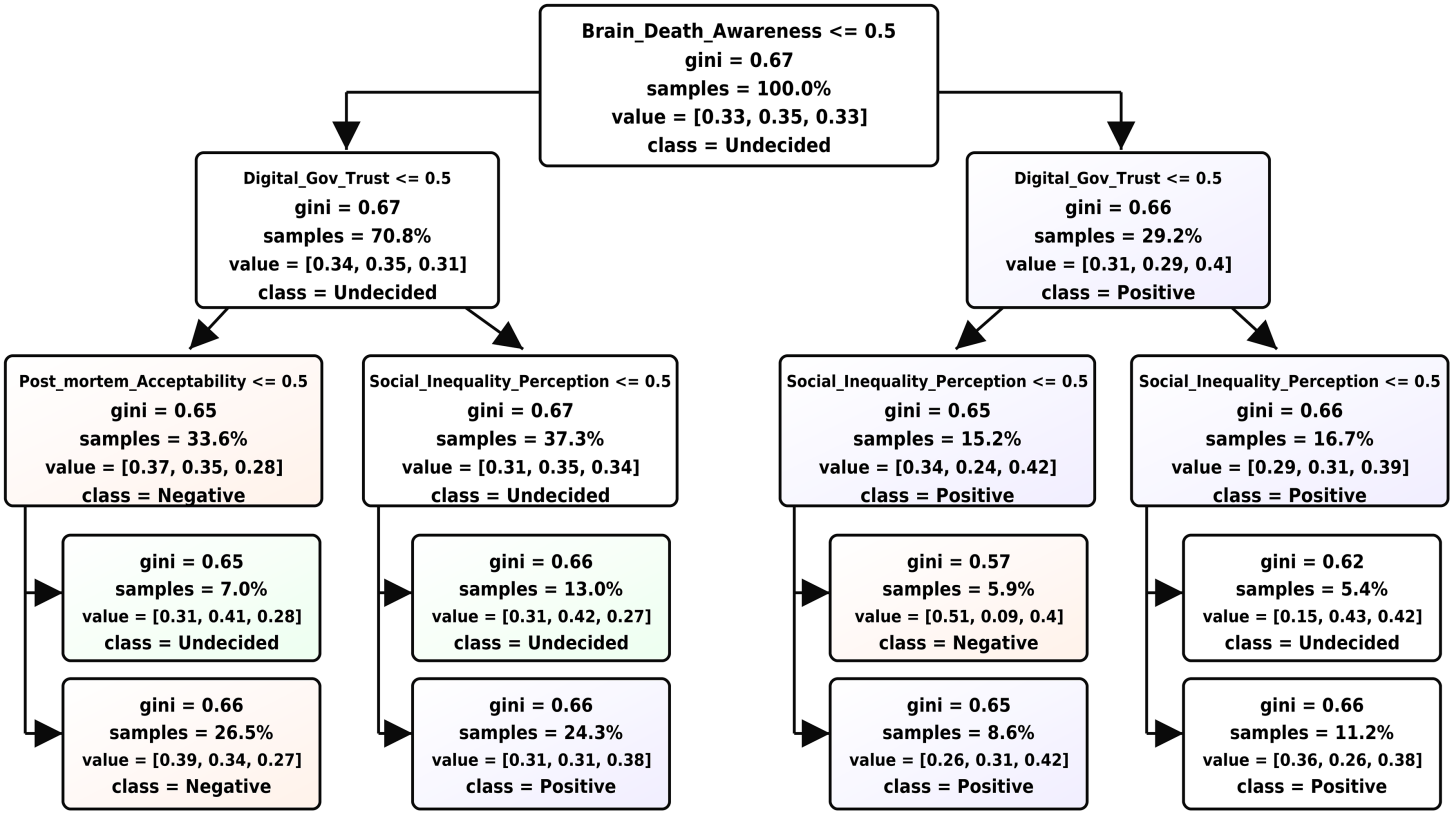
Annotated decision tree classifier illustrating hierarchical effects of brain-death awareness, digital government trust, social inequality perception, and post-mortem acceptability.

Overall, the findings indicate that attitudes toward deceased organ donation are shaped through the interaction of cognitive awareness, institutional trust, and ethical perceptions rather than through isolated demographic factors alone. Although the predictive performance of the model remained moderate, the analysis highlighted medical awareness and institutional confidence as potentially important factors associated with supportive attitudes toward deceased organ donation in Kazakhstan (see Figure 2).

The model identified Brain-Death Awareness as the fundamental root node of the entire system, suggesting that awareness of brain-death criteria may function as a primary cognitive threshold shaping attitudes toward post-mortem organ donation. The decision tree segmented respondents into distinct attitudinal clusters, demonstrating that the combination of higher medical awareness and stronger institutional trust was associated with the highest proportion of positive attitudes toward deceased organ donation. Model performance and parameter settings are summarized in Table 5. Performance metrics were calculated using the full analytical sample (*n* = 569), as no separate training-test split was applied due to the exploratory and interpretative nature of the model.

**Table 5:** Performance metrics of the decision tree classifier.

Metric	Precision	Recall	F1-score
Overall accuracy	–	–	0.37
Macro average	0.34	0.34	0.33
Weighted average	0.43	0.37	0.39

Model parameters	Value	Validation metrics	Value
Criterion	Gini	Mean CV accuracy	0.34
Max depth	3	Standard deviation	0.03
Root Gini index	0.66	–	–

Source: authors’ survey (2025).

Table 6 presents the confusion matrix of the decision-tree classifier and provides a descriptive overview of classification outcomes across attitudinal categories.

**Table 6:** Confusion matrix of the decision tree classifier.

Category	Predicted negative	Predicted undecided	Predicted positive	Recall (fullness)
Actual negative	50	29	51	38.50%
Actual undecided	32	36	40	33.30%
Actual positive	91	80	160	48.30%
Precision (accuracy)	28.90%	24.80%	63.70%	Total: 569

Source: authors’ survey (2025).

Despite the natural variability of socio-behavioral data, the model achieved a precision of 63.7% for the Positive category. Although the overall predictive performance remained modest, the model identified interpretable conditional relationships among awareness, institutional trust, and ethical perceptions. These findings support the use of the decision-tree framework as an exploratory analytical tool for visualizing hierarchical attitudinal structures rather than as a high precision predictive model. Additional validation details are provided in Appendix C.

## 4 Discussion

### 4.1 Interpretation of findings

The findings indicate that education was the most consistent predictor associated with awareness of deceased organ donation and lower belief in organ-trafficking narratives. Respondents with higher educational attainment were more likely to correctly identify legal and medical mechanisms of post-mortem donation and less likely to endorse misinformation-related beliefs concerning organ trafficking. These patterns may suggest that higher education contributes to greater health literacy and more informed engagement with transplantation-related information. In contrast, age differences were associated primarily with preferred communication channels rather than with statistically significant differences in trafficking related beliefs.

The observed gap between awareness (63%) and knowledge (21%) suggests that exposure to information does not necessarily translate into understanding. This distinction is critical, as informed attitudes toward organ donation depend not only on familiarity with the concept but also on accurate comprehension of its medical and legal criteria.

Given the cross-sectional nature of the study, all observed relationships should be interpreted as associations rather than causal effects. The analyses identify patterns of cooccurrence between sociodemographic characteristics and attitudes toward organ donation but do not allow conclusions about temporal ordering or causal mechanisms.

Cross-national descriptive comparisons presented in the study suggest that systems characterized by sustained public communication, institutional transparency, and coordinated awareness campaigns tend to demonstrate higher levels of post-mortem donation participation; however, these contextual observations were not included in the statistical modelling and should be interpreted as descriptive benchmarks rather than causal explanations. Overall, the findings highlight the importance of strengthening educational outreach, improving institutional transparency, and addressing misinformation dynamics as key components of strategies aimed at increasing public willingness to consent to organ donation.

The findings additionally suggest that religion in Kazakhstan may function less as an isolated socio-demographic characteristic and more as part of the broader informational and normative environment surrounding transplantation. Within the present study, religion-related response categories were primarily associated with communication channels and moral interpretations of deceased donation rather than with direct measures of individual religiosity. This pattern is consistent with broader post-Soviet contexts, where public attitudes toward healthcare practices are frequently shaped through institutional narratives, informal communication, and social trust.

Although theoretical and empirical research suggests that education influences attitudes toward organ donation through health literacy and institutional trust, formal mediation analysis was not conducted in this study due to the absence of validated multi-item scales for these constructs in the survey instrument. As a result, education was treated as a proxy for underlying informational and trust-related mechanisms. Future research should explicitly model these pathways using validated measures and longitudinal designs.

The adjusted analysis indicates that structural factors, particularly place of residence, play a more decisive role in registration of consent for post-mortem organ donation than individual sociodemographic characteristics. The observed residence effect may reflect differences in local trust environments, patterns of social participation, and institutional engagement rather than simple disparities in informational access alone. Respondents residing in smaller urban and rural settlements may experience stronger community-based normative structures and closer interpersonal trust networks, which could facilitate willingness to support socially framed health initiatives such as organ donation. While education and income demonstrated economically meaningful tendencies, their effects appeared to operate primarily through broader informational and trust-related mechanisms rather than as direct independent predictors. These findings are broadly consistent with international evidence highlighting the importance of institutional coordination, social trust, and public engagement in shaping donor-registration behavior.

Although this finding may initially appear counter-intuitive given the concentration of healthcare infrastructure and digital services in Almaty city, it may reflect broader differences in social cohesion and institutional trust rather than technical access alone. Residents of smaller urban and rural communities may be more embedded in interpersonal support networks and collective moral frameworks that facilitate participation in socially oriented health initiatives. By contrast, residents of large metropolitan environments may be more exposed to fragmented information ecosystems, institutional skepticism, and polarized public discourse surrounding transplantation.

At the same time, the comparatively weaker associations between place of residence and general awareness or trafficking-related beliefs suggest that the observed residence effect may reflect broader institutional and social participation mechanisms rather than differences in basic informational exposure alone.

The decision tree analysis confirms the limited explanatory power of individual sociodemographic variables in predicting attitudes toward deceased organ donation. Despite explicit modeling of non-linear interactions and hierarchical splits, the tree exhibited shallow depth and low discriminative performance. This finding aligns with the results of the multivariable logistic regression and suggests that individual-level characteristics alone are insufficient to explain registration behavior. The weak performance of the tree model also reflects limited statistical power for subgroup analyses, given the low prevalence of positive outcomes. Rather than undermining the results, this convergence across parametric and nonparametric approaches strengthens the conclusion that structural and institutional factors play a more decisive role than individual demographics.

The use of balanced class weights (class_weight = “balanced”) addressed outcome imbalance and ensured adequate representation of minority categories. The overall predictive performance of the model can be characterized as modest, with moderate cross-validated stability. The confusion matrix provides a descriptive overview of classification patterns rather than serving as evidence of model reliability.

Importantly, the decision-tree model was not intended to maximize predictive precision but rather to complement the regression analysis by visualizing conditional interaction structures among cognitive, institutional, and ethical determinants of donation attitudes. Whereas the logistic regression model identified independent demographic predictors of formal donor registration, the decision-tree approach illustrated how awareness, institutional trust, and perceptions of social inequality interact hierarchically in shaping supportive attitudes toward deceased organ donation. In this sense, the model contributes interpretative and exploratory value by revealing structurally relevant pathways that may not be fully captured through conventional regression-based analysis alone.

Although the overall predictive performance of the model was modest, the decision tree provides an interpretable exploratory structure that highlights conditional relationships among cognitive and institutional factors. The resulting tree structure is relatively simple, with primary splits driven by brain-death awareness and trust in digital government, followed by perceptions of social inequality and post-mortem acceptability. These variables demonstrate conditional associations, while their discriminative contribution remains moderate. The model should therefore be interpreted as an exploratory analytical framework that identifies potentially relevant attitudinal patterns without implying strong predictive precision or definitive policy conclusions.

### 4.2 Policy implications and future directions

The findings have several practical implications for organ donation policy and public communication in Kazakhstan. First, public campaigns should place greater emphasis on educational content: clear, accessible explanations of brain-death criteria, consent procedures, and organ-allocation rules can reduce the space for rumours and conspiracy narratives. Second, it is more effective to adapt communication channels than to radically change the core message. For younger audiences, concise formats on social media and other digital platforms are likely to be most effective; for older adults, communication through clinicians, television, and community media may be more appropriate; for middle-aged groups, information disseminated at workplaces, professional associations, and civic organisations can be particularly relevant.

Third, there is significant potential in building cross-sector coalitions. Partnerships between healthcare professionals, educators, and religious leaders can strengthen the moral and institutional legitimacy of deceased donation and help counter misinformation in different social groups. Regular, publicly accessible reports or dashboards on waiting lists, allocation criteria, and transplant outcomes could further normalise transparency and gradually build trust in the transplantation system.

This study also has limitations. The analysis is based on data from a single territorial context, namely the city of Almaty and the Almaty region, uses a cross-sectional design, and relies on self-reported attitudes, which may be influenced by social desirability. Future research should extend the sampling frame to other regions, and complement survey data with qualitative methods such as in-depth interviews or focus groups to explore the moral reasoning behind consent and refusal decisions.

The study did not include direct measures of religious affiliation or religiosity, which limited the possibility of assessing religion as an independent predictor of donation attitudes. Future research may benefit from incorporating multidimensional indicators of religiosity and institutional religious trust into predictive models of organ-donation behavior.

Comparative studies in other post-Soviet and majority-Muslim settings would help clarify how institutional trust, religious authority, and media environments interact in shaping attitudes toward deceased organ donation. In addition, due to the relatively low proportion of respondents willing to register formal consent, the categories of refusal and uncertainty were combined in the binary logistic regression model. This approach improved statistical stability but may limit the precision of interpretation regarding behavioral differences between hesitant and explicitly refusing respondents.

In this broader perspective, the finding that education-rather than age-is the most consistent predictor of informed and ethically grounded attitudes becomes especially important: it suggests that strengthening health literacy and institutional transparency is a more promising route to increasing consent than appeals to abstract “generational differences.”

### 4.3 Contextual benchmarking and institutional background

According to official data from the Republican Center for Coordination of Transplantation and High-Tech Medical Services, Ministry of Health of the Republic of Kazakhstan (2025), 4,226 patients-including 128 children-remain on the national waiting list for organ transplantation. Only 15% of all transplants are post-mortem; the rest rely on living donors (Table 7).

**Table 7:** Dynamics of organ transplantation in Kazakhstan, 2018–2024.

Year	Patients waiting	Deceased donors (%)	Living donors (%)
2018	3,220	12	88
2020	3,948	14	86
2022	4,106	15	85
2024	4,226	15	85

Source: republican center for transplantation (2025).

This pattern suggests that, despite the existence of formal legislative and institutional mechanisms regulating organ donation, public participation in post-mortem donation programs remains comparatively limited. These findings are consistent with previous studies conducted in post-Soviet healthcare contexts, where institutional distrust and fragmented communication environments continue to constrain donor registration and public engagement (Bolatov et al., 2025b).

**Figure 3:**
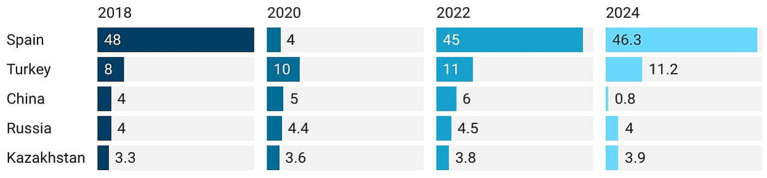
Donors per million population (2018–2024): Spain, Turkey, China, Russia, Kazakhstan.

The comparative framework used in this study suggests that public attitudes toward deceased organ donation are shaped not only by individual awareness but also by broader institutional and communication environments. Countries characterized by sustained public awareness strategies and higher institutional transparency tend to demonstrate higher levels of public engagement with post-mortem donation systems. In contrast, fragmented communication environments and lower institutional trust may contribute to the persistence of misinformation and lower readiness for formal donor registration. Within this broader context, the findings from Kazakhstan appear consistent with patterns observed in other transitional healthcare systems, where health literacy and institutional confidence remain closely interconnected (see Figure 3).

These contextual patterns support the interpretation that institutional trust, communication transparency, and health literacy may play an important role in shaping public engagement with post-mortem organ donation systems.

## 5 Conclusion

This study offers the first empirical sociological analysis of how sociodemographic factors shape public perceptions of deceased organ donation and organ trafficking in Kazakhstan.

Drawing on a quota-representative urban survey (*n* = 569) and combining Fisher’s Exact Test with an interpretable Decision Tree Classifier, we demonstrate that education rather than age is the most consistent predictor of informed and critical attitudes. Age mainly distinguishes preferred information channels (digital versus traditional media) rather than underlying beliefs. In this sense, attitudes toward organ donation emerge less as a generational clash of values and more as an issue of health literacy and the quality of institutional communication.

The cross-country comparison with Spain, Turkey, China, and Russia places Kazakhstan within a group of systems where formal legal frameworks have been created but public trust and communicative legitimacy remain fragile. Spain illustrates the impact of long-term, transparent coordination; Turkey shows how religious endorsement can support donation in morally sensitive contexts; China highlights the role of intensive, state-led media and literacy campaigns following governance reforms; Russia reflects challenges similar to those in Kazakhstan, with chronic deficits in trust. Taken together, these cases suggest that credible communication and moral legitimation are key levers for increasing participation in post-mortem donation.

Several practical implications follow from these findings. First, communication strategies should be education-focused, offering clear explanations of concepts such as brain death, consent procedures, and allocation rules in accessible language. Second, it is more effective to tailor channels than to radically change messages: concise digital formats for younger audiences, clinician- and community-based communication for older adults, and workplace or civic platforms for middle-aged groups. Third, cooperation between clinicians, educators, religious leaders, and policymakers can help strengthen legitimacy and counter misinformation. Regular publication of transparent statistics on waiting lists, allocation criteria, and outcomes would further normalize openness and support trust.

At the same time, the study has limitations. The data come from a single territorial setting, namely the city of Almaty and the Almaty region; the design is cross-sectional, and attitudes are measured through self-report. Future research should extend the analysis to other regions of Kazakhstan, include qualitative interviews to explore moral reasoning in greater depth, and use experimental or longitudinal approaches to test the effects of targeted health-literacy interventions. Comparative work with other post-Soviet and majority-Muslim contexts would help clarify how institutional trust, religious authority, and media ecosystems interact in shaping donation attitudes.

In sum, the article contributes to health sociology by empirically demonstrating that education represents an important determinant of attitudes toward deceased organ donation and by providing practical implications for public-health communication adapted to different informational environments. The findings suggest that strengthening health literacy and transparent institutional communication may represent one of the most promising approaches for increasing documented consent and reducing misinformation surrounding organ donation across generations in Kazakhstan.

While the findings demonstrate consistent associations, the cross-sectional design of the study calls for cautious interpretation with respect to causal direction.

## Data Availability

The original contributions presented in the study are included in the article/Supplementary material, further inquiries can be directed to the corresponding author.
