# Supplementary material for: Sociodemographic predictors of public attitudes toward deceased organ donation and organ trade in Kazakhstan
Source: Front Sociol. 2026 Jun 26;11:1755804. doi: 10.3389/fsoc.2026.1755804 (PMC13349392; doi:10.3389/fsoc.2026.1755804)
Supplement: Supplementary file 1 [file Supplementary_file_1.pdf]

Appendix A. Questionnaire.

Questionnaire

Dear respondents! Al-Farabi Kazakh National University aims to conduct a sociological analysis and research on the topic “Organ donation in Kazakhstan: sociological context of the study.”

Please read the questions and answer options provided, select the answer that suits you best, and circle its number. If none of the answers provided satisfy you, indicate your own option in the “other” column. This survey is completely anonymous, and all data will be used only in aggregate form after processing.

1. How old are you?
1) 18-35 years old
2) 36-44 years old
3) 45-59 years old
4) 60 years and older

2. What is your gender?
1) Male
2) Female

3. What is your social status?
1) Civil servant
2) Education worker
3) Healthcare worker
4) Entrepreneur
5) Unemployed
6) Service worker
7) Retired
8) Student
9) Other (please specify) ________________________________

4. Your education?
1) Secondary education
2) Secondary vocational education (college, technical school)
3) Incomplete higher education
4) Higher education

5. Place of residence
1) City of republican significance
2) City
3) District centers, village

6. Your monthly income
1) - 85,000-250,000 tenge
2) - 250,000-360,000 tenge
3) - 370,000-500,000 tenge
4) - over 500,000 tenge
5) - no income

7. Are you aware of organ donation and transplantation in Kazakhstan?
1) Yes, I am aware
2) Yes, I am somewhat aware
3) No, I am not aware (go to question 7)
4) Difficult to answer

7.1 From what sources are you aware of "organ donation"?
1) Internet (VK, Instagram, Facebook, Twitter)
2) Radio
3) Television
4) Workplace
5) Place of study
6) Life experience (family/friends/relatives/acquaintances)
7) Religious organizations
8) Other (please specify) ________________________________

7.2 In your opinion, how would you rate the process of providing recipients (patients who receive organ transplants) with potential donors in the Republic of Kazakhstan? (5 - highest, 1 - lowest).
5 -------------- 4 -------------- 3-------------- 2-------------- 1

7.3 Are you aware of the "waiting list for organ donors"?
1) Yes 2) No

8. In your opinion, what are the primary sources of information about organ donation and transplantation? (select no more than 3 options)
1) From the Ministry of Health of the Republic of Kazakhstan (reports, official documents, information)
2) Publications by researchers in the field of medicine
3) From the Republican Center for Coordination of Transplantation and High-Tech Medical Services
4) From special medical programs
5) From social videos, social advertising, the media
6) From religious organizations and their representatives
7) Annual lectures/presentations by transplant coordinators at workplaces/educational institutions
8) Other (please specify) ________________________________

9. Which of the following opinions on the acceptability of organ transplantation from one person to another in order to save their life would you be more likely to agree with? (select no more than 3 options)
1) Only one of a pair of organs (kidney, lungs, liver lobe, etc.) from a living donor should be used (without causing irreparable harm to the donor's health)
2) Deceased donation may be used with the consent of relatives who have been diagnosed with brain death.
3) If a person signs an agreement via e.gov during their lifetime to become a donor after their death, their organs may be used automatically, without the consent of their relatives
4) It is unacceptable to use the organs of one person to treat another—although this will save the life of the patient, it will cause irreparable harm to the donor's health.
5) The organs of people who have consented to euthanasia (the medical termination of the life of seriously ill patients) may be used after their death.
6) Organ donation is an unnatural process: everyone should live as long as they are meant to.

10. How willing would you be to become an organ donor if it were necessary to save the life of your children, parents, spouse, blood relative, or close friend?
1) I am always willing.
2) I feel ready
3) I am not morally ready
4) I am not ready at all
5) I find it difficult to answer

11. What would you do if one of your close relatives decided to become a donor for you if necessary?
1) I would accept the help, because he is my close relative and I would not feel indebted.
2) I would accept the help, and I would definitely repay them in the future.
3) I would refuse their organs, as I would feel indebted to them for the rest of my life.
4) I would refuse their organs, as I would not want their health to suffer because of me.
5) Other ____

12. Do you think there is organ trafficking on the black market in Kazakhstan?
1) Yes, I have come across such information on the internet
2) Yes, I have heard about the existence of special medical centers that perform illegal transplants.
3) Yes, I have heard from acquaintances who are on the organ waiting list.
4) No, according to Kazakhstani law, the sale and purchase of organs is illegal.
5) No, there is no organ trade in Kazakhstan.
6) No, this is a rumor spread in society to cause distrust of medicine.
7) Other (please specify) ________________________________

13. Do you think it is acceptable to donate organs during one's lifetime to unrelated persons on a commercial basis, for remuneration?
1) Yes, everyone can use their organs on a commercial basis
2) Yes, because it is better to have legal permission to sell than to perform transplants in unsanitary conditions and sell organs illegally.
3) Yes, donors who sell organs and recipients who buy organs cannot be punished because they are disabled.
4) People should be altruistic and donate organs for free.
5) Negative, because this situation leads to human trafficking.
6) Other (please specify) ________________________________

14. As a citizen of the Republic of Kazakhstan, would you register your consent to become a donor after death on the e.gov portal?
1) I would give my consent.
2) I would refuse to give my consent.
3) Not sure, need to think about it

15. In your opinion, what is the best way to register citizens' decisions to consent or refuse to consent to organ donation in Kazakhstan?
1) In an open form (in driver's licenses, donor cards, medical records)
2) In a closed form (e.gov.kz portal)
3) It doesn't matter (either open or closed form is acceptable)
4) It is not necessary to register this in any form
5) Other (please specify) ________________________________

16. In your opinion, is it acceptable to use organs from a deceased donor?
1) Yes, it is acceptable; it is better than causing irreparable harm to the health of a living donor
2) It is acceptable only in cases where an unpaired vital organ is needed (e.g., heart, pancreas)
3) It is not acceptable; it contradicts my religious beliefs (go to question 16)
4) It is not acceptable, as it contradicts my ethical and moral principles (go to question 16).

17.1 Do you support multi-organ donation (donation of more than one organ) in the case of a brain-dead donor?
1) I support it, as it can save several lives at once
2) I support it, as it is more important to save lives
3) I do not support it, as I believe it is wrong to remove all of the deceased's organs
4) I do not support it; it is only acceptable to take organs that cannot be obtained from a living person (heart, pancreas)

18. Do you agree that there is socio-economic inequality in our country due to the shortage of donors?
1) Rich people can immediately find a donor in foreign clinics and save lives in a short time.
2) A person with low social status is likely to die while waiting in line.
3) Kazakhstan can fully provide organ donation.
4) Any citizen of Kazakhstan can find a donor and undergo a transplant abroad at the expense of the state.

19. What social assistance, in your opinion, should the state provide to living donors in Kazakhstan? (select no more than 3 options)
1) Free life insurance should be provided.
2) The donor's weekly workload should be reduced (less than 8 hours per day) while maintaining the same salary.
3) State guarantees of social protection (benefits, pensions, social service benefits, etc.) should be provided.
4) Organ donation should be strictly voluntary and altruistic; no assistance should be provided.

20. What kind of assistance do you think should be provided to the family of an organ donor who became a donor after their death?
1) The donor's family should be given a large sum of money.
2) The donor should be posthumously awarded a state award, such as “Hero-Saviour of Lives”.
3) The donor's family should be entitled to free medical care for life.
4) The donor's family should be provided with state guarantees of social protection (benefits, pensions, other social and living benefits, etc.).
5) Organ donation should be strictly voluntary and altruistic; no assistance should be provided.

21. What measures do you think need to be taken to promote the normal perception and development of organ donation in society?
1) Social advertising, billboards, banners, word of mouth...
2) Celebration of “Donor Day” at the state level
3) Campaigning by religious organizations
4) Opening of centers for moral preparation for donation
5) Other (specify)____

A brain-dead donor is a person who has consented to post-mortem organ donation, whose brain has died but whose other organs are still functioning.

Appendix B. Logistic Regression - Specification and Code

This appendix provides the reproducible Python code used to estimate the multivariable logistic regression model with robust standard errors (HC0).

Statistical Software
Python 3.x
Libraries: pandas, numpy, statsmodels

Model Specification
Dependent variable:
consent (binary: 1 = consent registered, 0 = no consent)
Independent variables:
age (continuous), gender (binary), education, income, place of residence (categorical)
Estimation method:
Logistic regression with robust standard errors (HC0)

```
import pandas as pd
import numpy as np
import statsmodels.formula.api as smf

# Load data
data = pd.read_excel("survey_data.xlsx")

# Inspect data structure
print(data.info())
print(data.isnull().sum())

# Convert categorical variables
data['education'] = data['education'].astype('category')
data['residence'] = data['residence'].astype('category')
data['income'] = data['income'].astype('category')

# Recode gender (0 = male, 1 = female)
data['gender'] = data['gender'].map({1: 0, 2: 1})

# Sample size and event rate
N = len(data)
events = data['consent'].sum()
event_rate = events / N

print("Number of observations:", N)
print("Number of events:", events)
print("Event rate:", round(event_rate, 4))

# Logistic regression with robust standard errors (HC0)
model = smf.logit(
    'consent ~ age + gender + C(education) + C(residence) + C(income)',
    data=data
).fit(cov_type='HC0')

# Model summary
```

```
print(model.summary())

# Model fit statistics
print("Pseudo R²:", round(model.prsquared, 3))
print("LLR p-value:", round(model.llr_pvalue, 3))

# Table of coefficients (log-odds)
logit_table = pd.DataFrame({
    "Variable": model.params.index,
    "Coefficient (log-odds)": model.params.values,
    "Robust SE (HC0)": model.bse,
    "z-statistic": model.tvalues,
    "p-value": model.pvalues,
    "Lower 95% CI (log-odds)": model.conf_int()[0],
    "Upper 95% CI (log-odds)": model.conf_int()[1]
})

# Adjusted Odds Ratios (AOR)
aor_table = pd.DataFrame({
    "Variable": model.params.index,
    "AOR": np.exp(model.params),
    "Lower 95% CI": np.exp(model.conf_int()[0]),
    "Upper 95% CI": np.exp(model.conf_int()[1]),
    "p-value": model.pvalues
})

print(aor_table)
```

Appendix C. Decision Tree Validation

Figure C1. Annotated decision tree
This figure presents the annotated decision tree classifier constructed using cognitive, institutional, and ethical predictors of attitudes toward post-mortem organ donation.

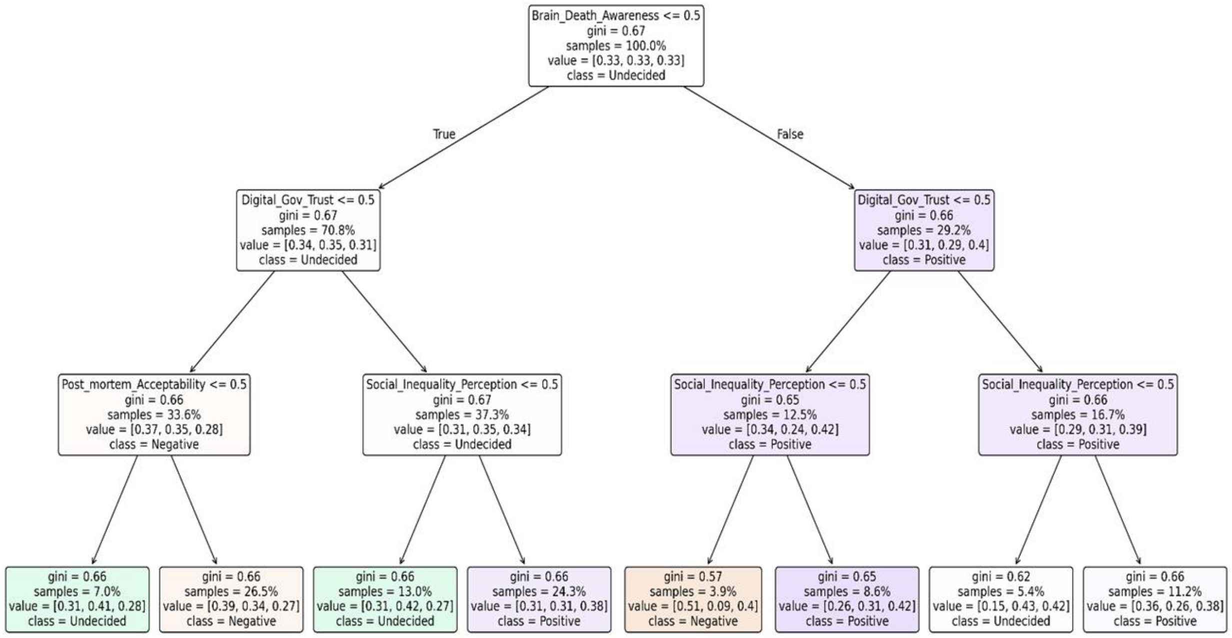

Figure C2. Confusion matrix of the decision tree classifier
This figure shows the confusion matrix summarizing the classification performance of the decision tree model across the three outcome categories (Negative, Undecided, Positive).

Category	Predicted Negative	Predicted Undecided	Predicted Positive	Recall (Fullness)
Actual Negative	50	29	51	*38.50%*
Actual Undecided	32	36	40	*33.30%*
Actual Positive	91	80	160	*48.30%*
Precision (Accuracy)	28.90%	24.80%	63.70%	Total: 569

Figure C3. Code used to generate the decision tree classifier
This code illustrates the main steps involved in data preprocessing, variable recoding, model specification, and estimation of the decision tree classifier.

```
import pandas as pd
import matplotlib.pyplot as plt
from sklearn.tree import DecisionTreeClassifier, plot_tree
from sklearn.metrics import confusion_matrix

# 1. DATA PREPROCESSING
df = pd.read_excel('survey_data.xlsx')
df.columns = df.columns.str.replace('"', '').str.replace('\n', '').str.strip()
```

```
rename_dict = {
    'Насколько вы готовы стать донором органа, если бы это было нужно для спасения жизни': 'Donation_Readiness',
    'egov': 'Digital_Gov_Trust',
    'Как вы понимаете понятие «смерть мозга': 'Brain_Death_Awareness',
    'Согласны ли вы с тем, что у нас в стране существует социально-экономическое неравенство в связи с дефицитом доноров': 'Social_Inequality_Perception',
    'Как по Вашему мнению, допустимо ли использование органов трупного донора': 'Post_mortem_Acceptability'
}
df = df.rename(columns=rename_dict)

# 2. MODEL SPECIFICATION
features = ['Digital_Gov_Trust', 'Brain_Death_Awareness', 'Social_Inequality_Perception', 'Post_mortem_Acceptability']
X = df[features]
y = df['Donation_Readiness']

clf = DecisionTreeClassifier(max_depth=3, criterion='gini', class_weight='balanced', random_state=42)
clf.fit(X, y)

# 3. RESULTS (TABLE 7)
y_pred = clf.predict(X)
cm = confusion_matrix(y, y_pred)
print(cm)
```

Appendix D. Variable operationalization and coding scheme

Construct, Analytical Variable	Questionnaire Items	Coding Logic	Variable Type
General awareness of deceased organ donation	Q7	1 = reports awareness of deceased organ donation; 0 = reports no awareness or uncertainty	Binary
Factual knowledge of donation criteria	Q7.3, Q9, Q15	1 = correctly identifies at least one legally valid mechanism of post-mortem donation or brain death; 0 = incorrect or uncertain response	Binary
Belief in organ trafficking	Q12	1 = believes illegal organ trade exists; 0 = does not believe or is uncertain	Binary
Registration readiness	Q14	1 = willing to register consent via e.gov; 0 = refusal or uncertainty	Binary
Digital government trust	Q8, Q14	1 = expresses trust in official institutional information sources and/or e.gov donor registration mechanisms; 0 = distrust or uncertainty	Binary
Ethical acceptability of deceased donation	Q9, Q10	1 = considers deceased organ donation morally acceptable; 0 = considers it unacceptable or expresses uncertainty	Binary
Perceived institutional inequality	Q12, Q13	1 = perceives unfair access, commercialization, or illegal transplantation risks; 0 = does not perceive such risks	Binary
Willingness toward organ donation (decision-tree outcome variable)	Main attitudinal outcome item	Negative / Undecided / Positive	Multiclass
