# Supplementary material for: Sociodemographic predictors of public attitudes toward deceased organ donation and organ trade in Kazakhstan
Source: Front Sociol. 2026 Jun 26;11:1755804. doi: 10.3389/fsoc.2026.1755804 (PMC13349392; doi:10.3389/fsoc.2026.1755804)
Supplement: Supplementary file 2 [file Table_1.DOCX]

Supplementary Material

Table 1 – Demographic profile of respondents. Source: authors’ survey, 2025.

| **Characteristic** | **Categories** | **% of sample (n = 581)** |
| --- | --- | --- |
| **Age** | 18–35 yrs (48.5 %); 36–44 yrs (19.8 %); 45–62 yrs (31.7 %) | 100 % |
| **Gender** | Women 54.9 %; Men 45.1 % | 100 % |
| **Education** | Higher 60 %; Incomplete higher 9.2 %; Vocational 13 %; Secondary 17 % | 99 % |
| **Income** | No income 25 %; ≤ 250 000 ₸ 33 %; 250–360 000 ₸ 24 %; > 500 000 ₸ 5 % | 87 % |

Table 2 – Comparative overview of organ-donation systems, 2018–2024. Sources: GODT 2024; WHO Transplant Observatory; Bolatov et al., 2025.

| **Country** | **Donors per million population (2024)** | **Main governance model** | **Public awareness trend** |
| --- | --- | --- | --- |
| **Spain** | 46.3 pmp | Centralized opt-out system with strong public trust | High engagement, stable positive media |
| **Turkey** | 11.2 pmp | Hybrid opt-in system with religious endorsement by Diyanet | Moderate awareness, religious support essential |
| **Russia** | 4.6 pmp | De facto opt-out, low institutional transparency | Low trust, limited campaigns |
| **China** | 6.8 pmp | State-regulated opt-in system (post-2015 reforms) | Rapid growth, strong media presence |
| **Kazakhstan** | 3.9 pmp | Opt-out by law, but opt-in in practice | Weak public trust, fragmented information |


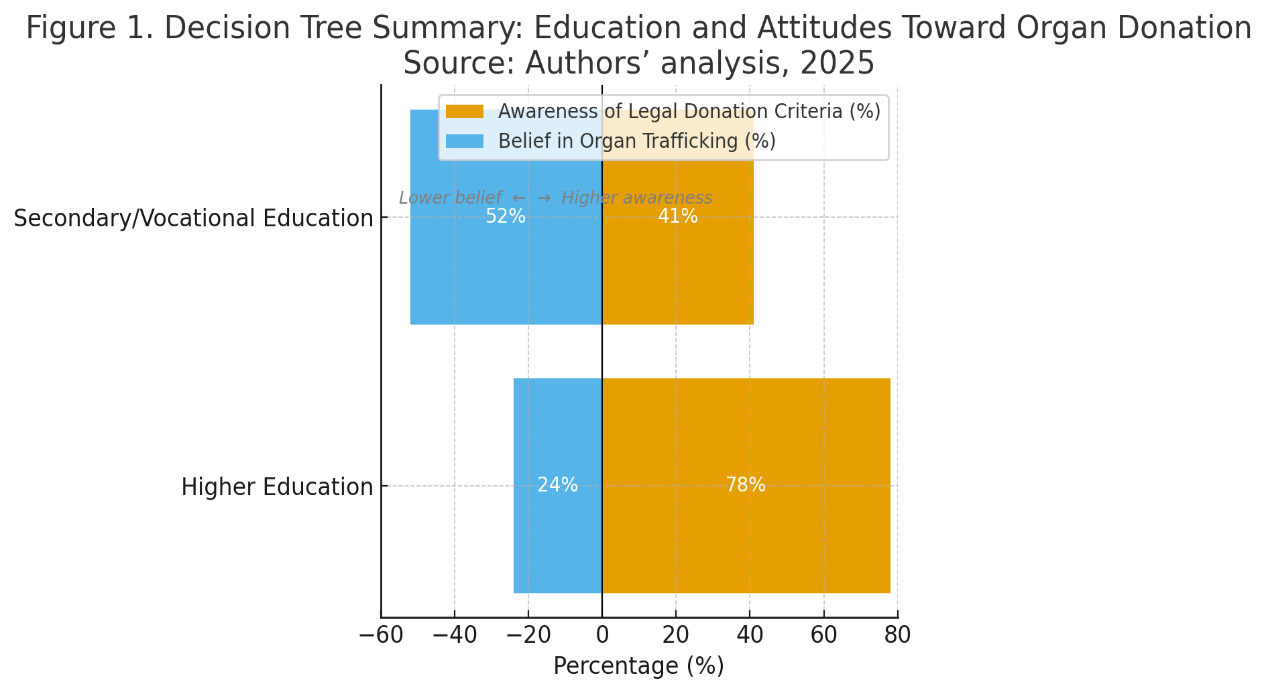


Figure 1 – Decision Tree of sociodemographic predictors of attitudes toward organ donation; Source: authors’ analysis, 2025.

Table 3 – Dynamics of organ transplantation in Kazakhstan, 2018–2024. Source: Republican Center for Transplantation (2025).

| **Year** | **Patients waiting** | **Deceased donors (%)** | **Living donors (%)** |
| --- | --- | --- | --- |
| 2018 | 3 220 | 12 | 88 |
| 2020 | 3 948 | 14 | 86 |
| 2022 | 4 106 | 15 | 85 |
| 2024 | 4 226 | 15 | 85 |

Table 4 – Awareness and belief levels by education. Source: authors’ survey (2025).

| **Education level** | **Awareness of legal donation criteria (%)** | **Belief in organ trafficking (%)** |
| --- | --- | --- |
| Higher | 78 | 24 |
| Incomplete higher | 59 | 37 |
| Vocational | 48 | 45 |
| Secondary | 41 | 52 |

Table 5 – Comparative overview of donor rates and trust levels.

| **Country** | **Donors pmp (2024)** | **Legal model** | **Public trust index*** | **Dominant information source** |
| --- | --- | --- | --- | --- |
| Spain | 46.3 | Opt-out, centralized coordination | High (0.82) | Public campaigns + media |
| Turkey | 11.2 | Opt-in with religious endorsement | Moderate (0.63) | Religious leaders + media |
| China | 6.8 | Opt-in (reformed system post-2015) | Rising (0.71) | State media + online portals |
| Russia | 4.6 | Opt-out de jure, opt-in de facto | Low (0.44) | Television + informal rumor |
| Kazakhstan | 3.9 | Opt-out by law, low public trust | Low (0.41) | Social media + TV |

Figure 2 – Donors per million population (2018–2024): Spain, Turkey, China, Russia, Kazakhstan.
